# BioCommons: a robust java library for RNA structural bioinformatics

**DOI:** 10.1093/bioinformatics/btab069

**Published:** 2021-02-03

**Authors:** Tomasz Zok

**Affiliations:** Institute of Computing Science, Poznan University of Technology, Poznan 60-965, Poland

## Abstract

**Motivation:**

Biomolecular structures come in multiple representations and diverse data formats. Their incompatibility with the requirements of data analysis programs significantly hinders the analytics and the creation of new structure-oriented bioinformatic tools. Therefore, the need for robust libraries of data processing functions is still growing.

**Results:**

BioCommons is an open-source, Java library for structural bioinformatics. It contains many functions working with the 2D and 3D structures of biomolecules, with a particular emphasis on RNA.

**Availability and implementation:**

The library is available in Maven Central Repository and its source code is hosted on GitHub: https://github.com/tzok/BioCommons

**Supplementary information:**

Supplementary data are available at *Bioinformatics* online.

## 1 Introduction

Structural biology and bioinformatics deal with studying structures of biological molecules at different levels of detail: primary, secondary, tertiary and quaternary structures. For each of them, various representations exist for saving structural information. They allow highlighting and analyzing different features of the structure.

The primary structure (sequence) of the molecule is most often encoded in FASTA format, applying a 4-letter alphabet (for DNA and RNA) or 20-letter (for proteins). Additional letters are allowed to generalize the description.

As for the secondary structure, especially in the case of RNA, it is rich in representations, e.g. dot-bracket notation, base-pair list or a graphical diagram. Only some of them allow to represent pseudoknot characteristics.

The 3D and 4D structures are most often saved as a list of atoms with their coordinates (PDB or PDBx/mmCIF files). However, other options exist such as the trigonometric representation in the form of a list of torsion angles.

Each structural level has associated bioinformatics libraries and tools, with preferable representations and formats of input/output data. Many of them do not cope with data reformatting, missing atoms, redundant or inconsistent data (e.g. mismatches in numbering), etc. The presented BioCommons library is facing many of such problems and tasks concerning the processing of structural data.

## 2 Materials and methods

**Fig. 1.**
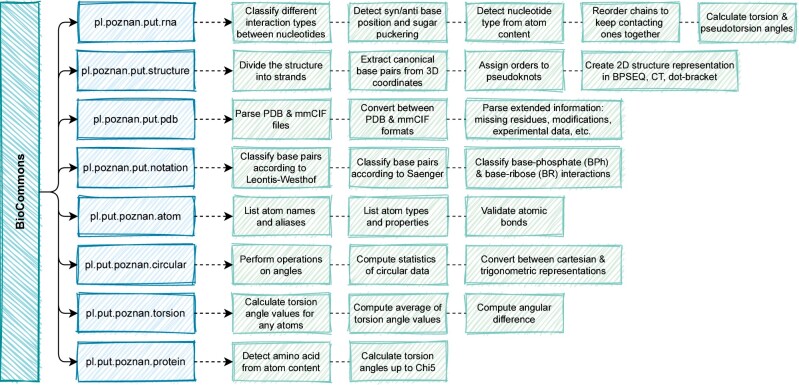
BioCommons is divided into packages (blue). Each package is responsible for a number of functions (green)

BioCommons is a Java library that brings together functions and data structures useful for structural bioinformaticians (Figure 1). It consists of eight main packages and four auxiliary ones containing together almost 400 classes and over 3000 methods (Table S1).

The library has a robust parser of PDB and mmCIF data. It detects aliases of atom names, includes missing residues in the analysis, and collects data from the header. The parser also employs a fuzzy detector of residue type from its heavy atom content. Additionally, it checks the present atomic bonds against a set of expected ones. The library contains a converter between PDB and mmCIF file formats. Due to PDB limits, i.e. five digits for atom number and four digits for residue number, BioCommons employs a smart resolution of conflicts. First, it creates a graph of contacting chains and retrieves all connected components. Then, BioCommons models format conversion as bin-packing problem by treating connected components as items and atom/residue counts as bin sizes. The library implements First-Fit heuristic to solve it. This way, even if a mmCIF structure has to be divided into several PDB structures, the library ensures that all contacting chains are stored together.

The set of functions for the 3D data analysis includes those related to trigonometric representation in torsion angle space. BioCommons supports all torsion angles for proteins and nucleic acids. For the latter, there are dedicated functions for pseudo-torsion angles *η*, *θ*, $\eta^{\prime }$ and $\theta^{\prime }$ (Keating *et al.*, 2011) as well as pseudo-phase pucker of the sugar ring (Saenger, 1984). Besides, the library enables statistical analysis of circular data and reconstruction of atomic coordinates from angle values.

BioCommons also allows a thorough analysis of nucleic acids secondary structure. First of all, it implements a geometric method of extracting canonical base pairs. They can be processed and stored in either BPSEQ, CT, or the extended dot-bracket notation. There is an optional graph-based optimization step toaccount for contacts between the chains. With dot-bracket notation, BioCommons can encode pseudoknots of any order (Zok *et al.*, 2018). The library contains implementations of a few heuristics and a dynamic programming algorithm (Smit *et al.*, 2008) for that purpose.

BioCommons can map the 2D and 3D levels of structure details. For example, one may analyze the 3D coordinates to find an interesting structural element in the 2D representation and then get back to its 3D model. Or one can quickly find out which secondary structure motif has the most modified residues and what is the distribution of their torsion angle values.

## 3 Discussion

According to the author’s knowledge, one Java library for bioinformatics, BioJava (Lafita *et al.*, 2019), has already been presented. It has a dedicated community, a mature codebase, and a variety of functions for multiple purposes. Like many other libraries and tools in the field, BioJava focuses on proteins—many of its classes and methods assume the data under consideration describe a protein structure. BioCommons complements this resource, primarily with functions tailored to RNAs, and taking into account the unique properties of their folds. The library is also compatible with bidirectional converters for selected structural objects (e.g. atoms).

## 4 Conclusion

BioCommons was developed for many years. Its components were created during the author’s work on the projects from the RNApolis suite (Szachniuk, 2019). The functionality of the library had practical application for torsion angles analysis, 3D and 2D data mapping, pseudoknot handling, nucleobase remodeling and many others (Table S2).

Many structural biologists and bioinformaticians, especially those focused on RNA molecules, may benefit from this library. It offers unique functionality not only in the Java ecosystem, such as its handling of pseudoknots of any order. BioCommons can robustly handle many structure representations and automatically fix common problems. Its source code is freely available, and the compiled versions are deployed to Maven Central Repository.

## Supplementary Material

btab069_Supplementary_Data
