# Supplementary material for: BioCommons: a robust java library for RNA structural bioinformatics
Source: Bioinformatics. 2021 Feb 3;37(17):2766–7. doi: 10.1093/bioinformatics/btab069 (PMC8428578; doi:10.1093/bioinformatics/btab069)
Supplement: btab069_Supplementary_Data [file btab069_supplementary_data.pdf]

BioCommons: a robust Java library for RNA structural bioinformatics

Supplementary Materials

Tomasz Zok

3D and 2D structure of RNAs

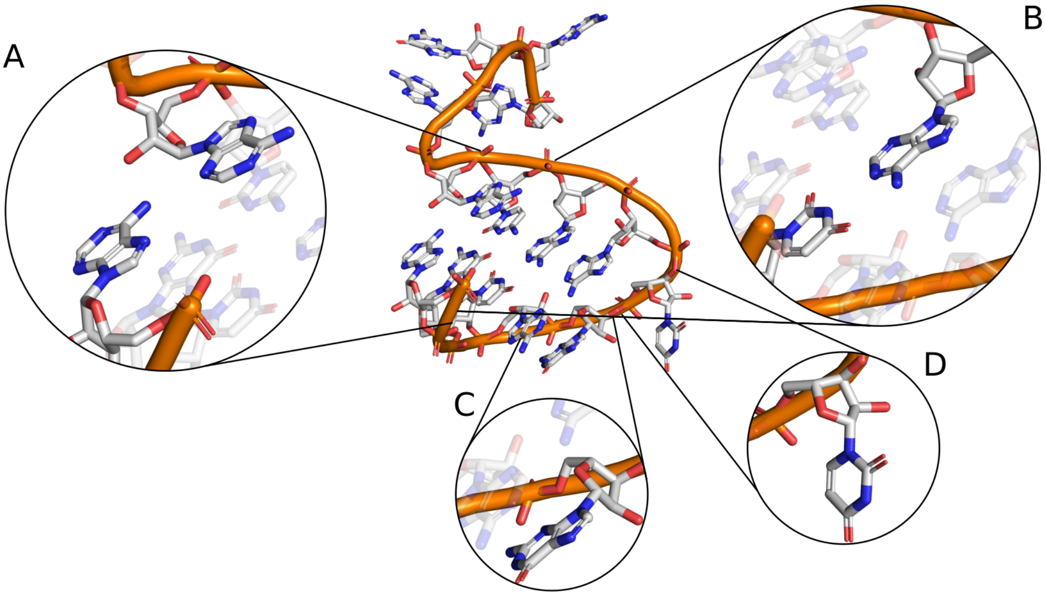

Figure 1: A fragment of bat influenza viral RNA (from pdb id: 6T0R) showing: (A) a non-canonical base pair, (B) a canonical base pair, (C) a nucleotide in syn conformation, and (D) a nucleotide in anti conformation.

Experimentalists and modellers store the 3D structure of RNA molecules in PDB or PDBx/mmCIF formats. Both consist of, in general, headers with metadata and a list of atom coordinates. Additional information about the 2D structure usually requires further processing by external tools. BioCommons allows to extract and interpret many features of the 2D structure from the 3D coordinates (see Figure 1), such as base pairs (canonical and non-canonical), base-phosphate and base-ribose interactions, stacking interactions, and nucleobase conformations (syn or anti).

Modified residues

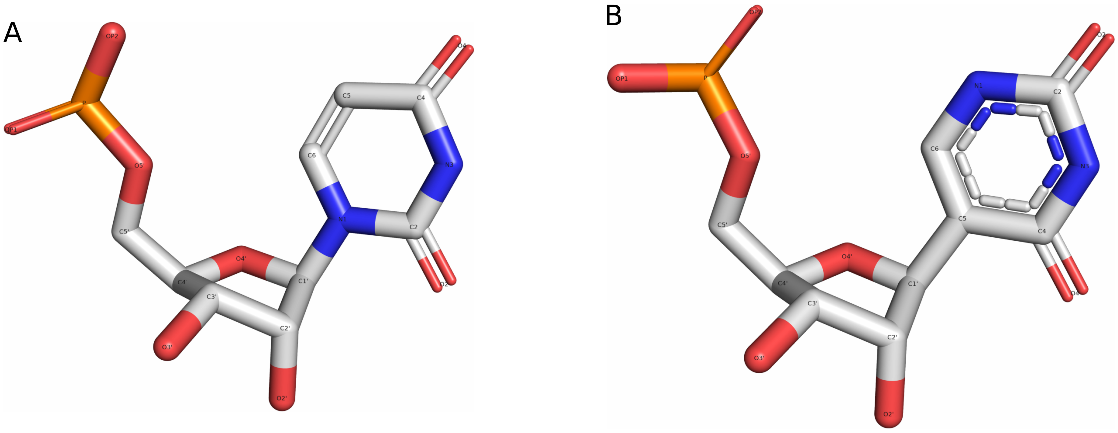

Figure 2: (A) uracil, (B) pseudouridine. Both nucleobases have the same heavy atom content, but (B) should be detected as a modified (A).

BioCommons can parse and process information about modified residues. It also employs a fuzzy detector of residue type, which comes in handy when working with 3D models generated in silico without any header information.

For example, BioCommons detects that residue A.10 in 1EHZ structure is modified guanine. The parser sets its name to 2MG, and the detector correctly marks that the heavy atom content is different than expected.

Even for A.16 – a dihydrouridine with additional hydrogens – and for A.39 – a pseudouridine, i.e. an isomer of uridine – the parser correctly marks the residues as modified, despite the heavy atom content is the same as in an unmodified uridine. The detector is sensitive to all atoms, so modifications to backbone or ribose also get caught. Of course, the same mechanism works for proteins, e.g. S.169 in 148L is correctly determined to be 2,6-diaminopimelic acid – a modified lysine.

Missing and unknown residues

The other distinguishing feature of BioCommons is its handling of missing residues. Information about them is available in the headers with details about the sequence of the missing parts. BioCommons reads these headers and embeds the information in its data structures.

A bit different case is with UNK residues (e.g. 3KFU), for which both the sequence and electron density maps are uncertain. BioCommons handles these as well.

```
>strand_A
aGCGCCuGGACUUAAAGCCAUUGCACU
..((((.((((((((((((..------
>strand_B
CCGGCUUUAAGUUGACGAGGGCAGGGUUuAUCGAGACAUCGGCGGGUGCCCUGCGGUCUUCCUGCGACCGUUAGAGGACU
GGuAAAACCACAGGCGACUGUGGCAUAGAGCAGUCCGGGCAGGAA
--)))))))))))..(((...[[[[[[...)))......)))))...]]]]]][[[[[.((((((]]]]].....(((((
(......((((((....)))))).......))))))..)))))).
```

Figure 3: A multi-chain, secondary structure (pdb id: 2Z74). Missing residues shown in red. Pseudoknots shown in blue.

Misformatted data

```
ATOM      2  P     G A   1      50.626  49.730  50.573  1.00100.19           P
ATOM      3  OP1   G A   1      49.854  48.893  49.562  1.00100.19           O
ATOM      4  OP2   G A   1      52.137  49.542  50.511  1.00 99.21           O
-------------------------------------------------------------------------------
ATOM     33 P    A       2       2.226   3.609   0.787  1.00  0.00           P
ATOM     34 OP1  A       2       0.898   4.237   0.641  1.00  0.00           O
ATOM     35 OP2  A       2       3.131   3.426  -0.364  1.00  0.00           O
```

Figure 4: An example of well-formatted (top) and misformatted (bottom) lines in PDB format. The atom name (green box) cannot be left-aligned according to the specification. A similar problem occurs for residue name (orange box). The chain identifier (purple box) cannot be blank.

Furthermore, BioCommons parser is robust in the face of misformatted data. The PDB format defines the following, among others: (source: https://www.wwpdb.org/documentation/file-format-content/format33/sect9.html#ATOM)

1. Alignment of one-letter atom name such as C starts at column 14, while two-letter atom name such as FE starts at column 13.
2. Non-blank alphanumerical character is used for chain identifier.
3. Columns 79 - 80 indicate any charge on the atom, e.g., 2+, 1-. In most cases, these are blank.

Many software tools which generate 3D models do not respect these rules. For example, PDB files generated with certain force-fields violate rule 1 by aligning atom names' to the left and rule 2 by using the blank character for chain identifier. Others end the line before 79 characters (violation of rule 3).

Residue order

The PDB specification requires to store lines describing atoms ordered according to increasing residue numbers. Even when this is true, a robust library needs to analyze

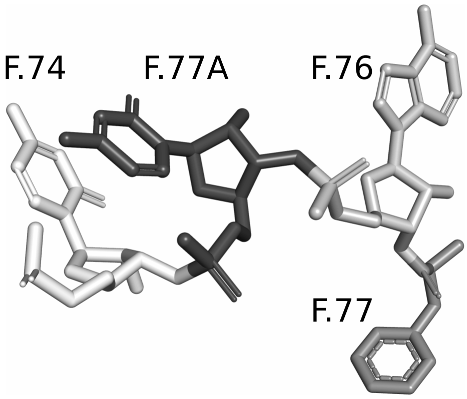

Figure 5: The last four residues of chain F in 1OB5 structure. Colors from light to dark represent the order of residues in the file. However, connections between residues are arranged in a different order.

the data to find the correct order as shows the example of 1OB5 structure. In the file, the numbers of the last residues in chain F are 74, 76, 77, 77A. Residue 75 is marked as missing in the headers. However, looking at the 3D coordinates reveals that the actual chain goes along 74, 77A, 76, 77. There is no gap in the chain as one would expect from the information in the headers. Additionally, the connections between residues appear in a different order than in the file. BioCommons correctly analyzes structures like that, which is crucial for i.a. torsion angles calculation.

Parsing time

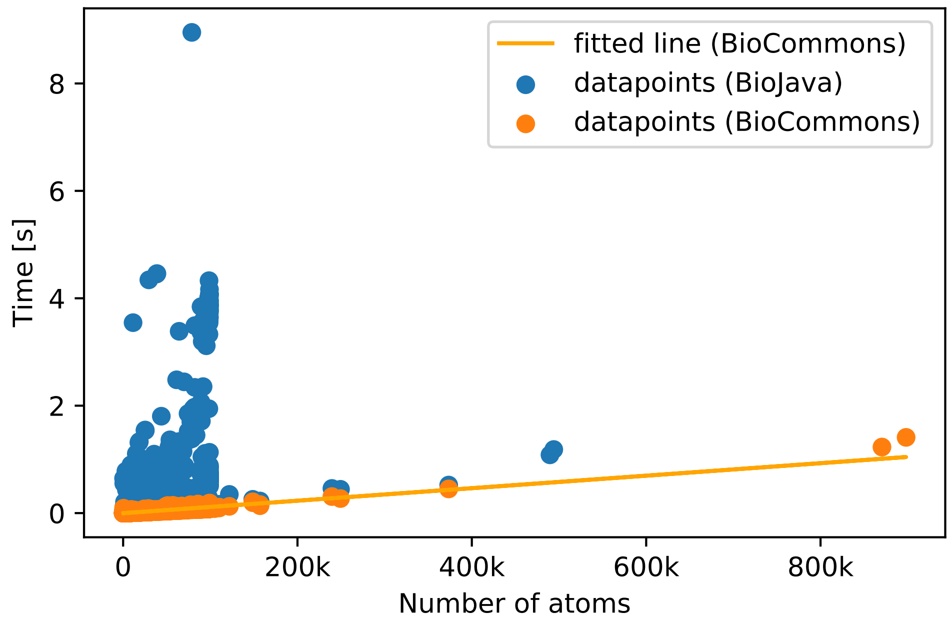

Figure 6: Parsing time of 11k+ nucleic acid structures in PDB format by BioCommons and BioJava.

Despite the overhead required for robust data handling, BioCommons is extremely fast to parse and process raw data. As of 2020-12-10, there are more than 11 thousands of PDB structures marked as nucleic acid (alone or in complex with a protein). In a computational experiment, BioCommons and BioJava were used to measure parsing time of all of them. The largest structure stored in PDB format (id: 6WLN) has 80 models with a total of almost 28k residues having 900k atoms. BioCommons required just 1.4 seconds to parse this file on a Linux machine with Intel® Core™ i7-2600K CPU @ 3.40GHz and 16 GB RAM. For this structure, BioJava returned an object consisting in sum of 15k residues having 494k atoms (when 6WLN is parsed by BioJava from mmCIF format, the number of residues and atoms are correct). In general, the parsing time by BioCommons library grows linearly with the number of atoms in the structure and even the largest ones can be processed quickly.

Classes and methods count

Table 1: BioCommons library in numbers

	Classes	Generated classes	**Total**	Methods	Generated methods	**Total**
`pl.poznan.put.atom`	6	2	**8**	15	22	**37**
`pl.poznan.put.circular`	14	10	**24**	83	110	**193**
`pl.poznan.put.constant`	3	0	**3**	10	0	**10**
`pl.poznan.put.interfaces`	5	0	**5**	10	0	**10**
`pl.poznan.put.notation`	10	0	**10**	30	0	**30**
`pl.poznan.put.pdb`	45	36	**81**	355	564	**919**
`pl.poznan.put.protein`	29	42	**71**	131	252	**383**
`pl.poznan.put.rna`	24	20	**44**	87	131	**218**
`pl.poznan.put.structure`	43	47	**90**	324	721	**1045**
`pl.poznan.put.torsion`	14	10	**24**	71	132	**203**
`pl.poznan.put.types`	4	4	**8**	13	51	**64**
`pl.poznan.put.utility`	14	5	**19**	62	65	**127**
Total	211	176	**387**	1191	2048	**3239**

Practical applications

Tool name	URL	Used in
MCQ4Structures	github.com/tzok/mcq4structures	Zok et al. (2014); Wiedemann et al. (2017); Magnus et al. (2020)
RNApdbee	rnapdbee.cs.put.poznan.pl	Antczak et al. (2014); Antczak et al. (2018); Zok et al. (2018)
RNAvista	rnavista.cs.put.poznan.pl	Rybarczyk et al. (2015); Antczak et al. (2019)
RNAfitme	rnafitme.cs.put.poznan.pl	Zok et al. (2015); Antczak et al. (2018)

BioCommons was developed for many years. Its components were created during the implementation of tasks appearing during the author's work on the projects from the RNApolis suite (Szachniuk, 2019). For example, for MCQ4Structures (Zok *et al.*, 2014; Wiedemann *et al.*, 2017), a set of functions was designed to compare biomolecules in torsion angle space. They have also been used in the assessment of RNA 3D structures predicted within RNA-Puzzles contest (Magnus *et al.*, 2020; Miao *et al.*, 2020). In RNApdbee (Antczak *et al.*, 2014; Antczak *et al.*, 2018; Zok *et al.*, 2018) and RNAvista (Rybarczyk *et al.*, 2015; Antczak *et al.*, 2019), the library's components formed a mapping layer between 2D and 3D structure data, especially in terms of pseudoknot handling. BioCommons also played a crucial role in the preparation of RNA conformer library (Zok *et al.*, 2015) for nucleobase and nucleoside remodeling within RNAfitme (Antczak *et al.*, 2018).

References

RNA-Puzzles Toolkit: A Computational Resource of RNA 3d Structure Benchmark Datasets, Structure Manipulation and Evaluation Tools. Marcin Magnus, Maciej Antczak, Tomasz Zok, Jakub Wiedemann, Piotr Lukasiak, Yang Cao, Janusz M. Bujnicki, Eric Westhof, Marta Szachniuk, Zhichao Miao. **Nucleic Acids Res**. 2020. *48*:576–588. doi:10.1093/nar/gkz1108

RNA-Puzzles Round IV: 3d Structure Predictions of Four Ribozymes and Two Aptamers. Zhichao Miao, Ryszard W. Adamiak, Maciej Antczak, Michal J. Boniecki, Janusz Bujnicki, Shi-Jie Chen, Clarence Yu Cheng, Yi Cheng, Fang-Chieh Chou, Rhiju Das, Nikolay V. Dokholyan, Feng Ding, Caleb Geniesse, Yangwei Jiang, Astha Joshi, Andrey Krokhotin, Marcin Magnus, Olivier Mailhot, Francois Major, Thomas H. Mann, Pawel Piatkowski, Radoslaw Pluta, Mariusz Popenda, Joanna Sarzynska, Lizhen Sun, Marta Szachniuk, Siqi Tian, Jian Wang, Jun Wang, Andrew M. Watkins, Jakub Wiedemann, Yi Xiao, Xiaojun Xu, Joseph D. Yesselman, Dong Zhang, Yi Zhang, Zhenzhen Zhang, Chenhan Zhao, Peinan Zhao, Yuanzhe Zhou, Tomasz Zok, Adriana Zyla, Aiming Ren, Robert T. Batey, Barbara L. Golden, Lin Huang, David M. Lilley, Yijin Liu, Dinshaw J. Patel, Eric Westhof. **RNA**. 2020. *26*(*8*):982–995. doi:10.1261/rna.075341.120

RNAvista: A Webserver to Assess RNA Secondary Structures with Non-Canonical Base Pairs. Maciej Antczak, Marcin Zablocki, Tomasz Zok, Agnieszka Rybarczyk, Jacek Blazewicz, Marta Szachniuk. **Bioinformatics**. 2019. *35*:152–155. doi:10.1093/bioinformatics/bty609

RNApolis: Computational Platform for RNA Structure Analysis. Marta Szachniuk. **FCDS**. 2019. *44*:241–257. doi:10.2478/fcds-2019-0012

New Algorithms to Represent Complex Pseudoknotted RNA Structures in Dot-Bracket Notation. Maciej Antczak, Mariusz Popenda, Tomasz Zok, Michal Zurkowski, Ryszard W Adamiak, Marta Szachniuk. **Bioinformatics**. 2018. *34*:1304–1312. doi:10.1093/bioinformatics/btx783

RNApdbee 2.0: Multifunctional Tool for RNA Structure Annotation. Tomasz Zok, Maciej Antczak, Michal Zurkowski, Mariusz Popenda, Jacek Blazewicz, Ryszard W Adamiak, Marta Szachniuk. **Nucleic Acids Res**. 2018. *46*:W30–W35. doi:10.1093/nar/gky314

LCS-TA to Identify Similar Fragments in RNA 3d Structures. Jakub Wiedemann, Tomasz Zok, Maciej Milostan, Marta Szachniuk. **BMC Bioinformatics**. 2017. *18*:456. doi:10.1186/s12859-017-1867-6

New in Silico Approach to Assess RNA Secondary Structures with Non-Canonical Base Pairs. Agnieszka Rybarczyk, Natalia Szostak, Maciej Antczak, Tomasz Zok, Mariusz Popenda, Ryszard W. Adamiak, Jacek Blazewicz, Marta Szachniuk. **BMC Bioinformatics**. 2015. *16*:276. doi:10.1186/s12859-015-0718-6

Building the Library of RNA 3d Nucleotide Conformations Using Clustering Approach. Tomasz Zok, Maciej Antczak, Martin Riedel, David Nebel, Thomas Villmann, Piotr Lukasiak, Jacek Blazewicz, Marta Szachniuk. **Int J Appl Math Comput Sci**. 2015. *25*:689–700. doi:10.1515/amcs-2015-0050

RNApdbee - a Webserver to Derive Secondary Structures from Pdb Files of Knotted and Unknotted RNAs. Maciej Antczak, Tomasz Zok, Mariusz Popenda, Piotr Lukasiak, Ryszard W. Adamiak, Jacek Blazewicz, Marta Szachniuk. **Nucleic Acids Res**. 2014. *42*:W368–W372. doi:10.1093/nar/gku330

MCQ4Structures to Compute Similarity of Molecule Structures. Tomasz Zok, Mariusz Popenda, Marta Szachniuk. **Cent Eur J Oper Res**. 2014. *22*:457–473. doi:10.1007/s10100-013-0296-5
